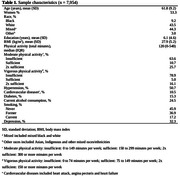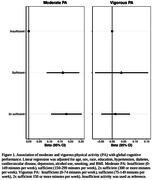# Association of moderate and vigorous physical activity with cognitive performance: results from the ELSI‐Brasil Study

**DOI:** 10.1002/alz.087567

**Published:** 2025-01-09

**Authors:** Ingryd Mayara Nascimento Martins‐Pais, Wendell Lima Rabelo, Naomi Vidal‐Ferreira, Cleusa P Ferri, Claudia Kimie Suemoto, Natalia G Gonçalves

**Affiliations:** ^1^ Universidade de Sao Paulo, Sao Paulo, SP Brazil; ^2^ Universidade Federal de São Paulo, São Paulo, SP Brazil; ^3^ Amazonia Adventist College, Benevides, PA Brazil; ^4^ Universidade Federal de São Paulo (UNIFESP), São Paulo, São Paulo/SP Brazil; ^5^ University of São Paulo Medical School, São Paulo, São Paulo Brazil; ^6^ University of São Paulo Medical School, São Paulo, SP Brazil

## Abstract

**Background:**

Moderate physical activity (PA) is considered one of the main protective factors for the risk of dementia. It is estimated that 1.6% of dementia cases worldwide could be prevented with increases in physical activity. However, there is little evidence of the association between vigorous physical activity and cognitive performance. Therefore, this study aimed to investigate the association between moderate and vigorous physical activity and cognitive performance in older adults from the Brazilian Longitudinal Study of Aging (ELSI‐Brasil).

**Method:**

The sample included 9,412 adults 50 years or older from the Brazilian Longitudinal Study of Aging. Cognitive performance evaluated the memory, temporal orientation, and verbal fluency domains. A global composite z‐score was derived from the tests. PA was assessed using the International Physical Activity Questionnaire. We used linear regression models to verify the association of moderate and vigorous physical activity with cognitive performance.

**Result:**

After exclusions, a total of 7,954 participants remained. Mean age was 61.8±9,2 years, 61.8% were women, 44.3% were mixed, 36.4% practiced at least 150 minutes of moderate physical activity, and 21.1% practiced at least 75 minutes of vigorous physical activity (Table 1). Compared to participants who did not meet the guideline for moderate PA (less than 150 minutes/week), those who met the guidelines (150 to 299 min/week) and those who performed more than 2x the recommended amount of moderate PA (300 minutes or more/week) had better global cognitive performance (β = 0.174, 95% CI = 0.097;0.250, p<0.001; β = 0.196, 95% CI = 0.124;0.268, p<0.001, respectively) (Figure 1). We found no association between vigorous PA and cognitive performance (Figure 1).

**Conclusion:**

While performing 150 minutes/week or more of moderate PA was associated with better cognitive performance, there was no additional benefit of vigorous PA for cognitive performance.